# Co-infection with dual *Streptococcus pneumoniae* serotypes as a cause of pediatric bacterial meningitis in Iran: a multi-center cross-sectional study

**DOI:** 10.1186/s12879-022-07606-w

**Published:** 2022-07-18

**Authors:** Sedigheh Rafiei Tabatabaei, Ahmadreza Shamshiri, Leila Azimi, Ali Nazari-Alam, Abdollah Karimi, Seyed Alireza Mirjavadi, Marjan Tariverdi

**Affiliations:** 1 Pediatric Infections Research Center, Research Institute for Children’s Health, Shahid Beheshti University of Medical Sciences, Tehran, Iran; 2 Department of Epidemiology and Biostatistics, School of Public Health, Tehran University of Medical Sciences, Tehran, Iran; 3 Infectious Diseases Research Center, Kashan University of Medical Sciences, Kashan, Iran; 4 17th Shahrivar Children’s Hospital, Guilan University of Medical Sciences, Guilan, Iran; 5 Department of Pediatric Infectious Diseases, Faculty of Medicine, Children’s Clinical Research Development Center, Hormozgan University of Medical Sciences, Bandar Abbas, Iran

**Keywords:** Bacterial meningitis, Co-infection, Dual infection, *Streptococcus pneumoniae*, Serotyping, PCR

## Abstract

**Background:**

Meningitis is considered a life-threatening infection with high mortality all over the world. *Hemophilus influenzae* (*H. influenzae*) and *Streptococcus pneumoniae* (*S. pneumoniae*) are regarded as the two most common infectious agents causing bacterial meningitis. This study aimed to identify *H. influenzae* and *S. pneumoniae* serotypes in blood and cerebrospinal fluid (CSF) of pediatric patients with meningitis, using polymerase chain reaction (PCR).

**Methods:**

This multi-center cross-sectional study included 284 children with suspected meningitis referred to 4 target hospitals. Overall, 412 samples (128 blood and 284 CSF samples) were obtained from the patients from November 14, 2016 to November 15, 2017. The extracted DNA was examined using multiplex real time PCR to screen for *S. pneumoniae* and *H. influenzae*. *S. pneumoniae* serotyping was also done by multiplex PCR.

**Results:**

Out of 284 CSF specimens, 22 were positive for *ply S. pneumoniae*. Of 20 DNA samples meeting the Quality Control (QC) standards for serotyping, 7 (35%), 6 (30%), 2 (10%), 2 (10%), 2 (10%), 1 (5%), 1 (5%), 1 (5%), 1 (5%) and 1 (5%) were positive for serotypes 3, 11A, 6A, 14, 7C, 23F, 23B, 19A, and 19F and 5, respectively. Overall, nine samples were positive for two serotypes, of whom 3 and 11A were the most common from Tehran province. Of note, one of these CSF samples showed a new co-infection with serotypes 7C and 14. Also, 6 samples (30%) were positive for *H. influenzae* detected by *bexA* primer. None of the blood samples were positive for *S. pneumoniae* or *H. influenzae*.

**Conclusion:**

Co-infection with *S. pneumoniae* serotypes can occur in bacterial meningitis and it might be missed if all serotypes are not evaluated in CSF specimens.

## Introduction

Reports indicate that 125,000 infants and young children are affected by meningitis annually, of whom 96% are in developing countries including Iran [1]. The most common bacterial agents causing meningitis in children are *Neisseria meningitidis *(*N. meningitidis*), *Haemophilus influenzae *(*H. influenzae*) and *Streptococcus pneumoniae *(*S. pneumoniae*). The mortality rate is more than 50% and 25–50% of the survivors suffer from serious neurological complications, including epilepsy, mental retardation, and sensorineural hearing loss [2, 3]. Lumbar puncture and cerebrospinal fluid (CSF) examination is the gold standard for the diagnosis of meningitis and should be performed for all suspicious cases of meningitis. The CSF specimen can be evaluated using different methods, including Gram staining, CSF differential cell count, measurement of protein and glucose concentrations, bacterial culture, latex agglutination, and polymerase chain reaction (PCR) [4–7]. In developing countries, Gram staining and bacterial culture are being used as routine standards for the confirmation of meningitis; however, these methods are time-consuming and have low sensitivity and specificity [4, 8]. Moreover, bacterial culture, the reference method for the diagnosis and determination of antibiotic susceptibility, takes at least two days and is not very sensitive; only 25% of bacterial meningitis cases yield positive cultures [3]. PCR plays an important role in identifying bacteria in bacterial meningitis and it is recommended when Gram staining and bacterial culture cannot confirm the disease. The main advantages of PCR are not requiring live bacteria and high sensitivity; nevertheless, PCR is not a routine diagnostic test in bacterial meningitis [9, 10]. Therefore, identifying common serotypes of the causative agents in a population may be helpful to tailor specific vaccines to each population and this will affect the national vaccination program and costs [11].

The pentavalent vaccine which protects against *H. influenzae* in addition to 4 other diseases (diphtheria, pertussis, tetanus, and hepatitis B), has been added to the national vaccination program in Iran since 2015. It appears that *H. influenzae* will probably be excluded from the list of major pathogens causing meningitis in Iran [12]. In previous studies, *S. pneumoniae* and *H. influenzae* were reported to be the major causes of meningitis in Iran, while infection with Enterobacteriaceae *spp.*, *N. meningitides* and group B streptococcus were rarely reported in subjects with meningitis [13–16]. In this study, we aimed to determine the *S. pneumoniae* and *H. influenzae* serotypes responsible for bacterial meningitis in a population of Iranian children.

## Materials and methods

### Study population and ethical considerations

The study was approved by the Ethics Committee of Shahid Beheshti University of Medical Sciences (IR.SBMU.RETECH.REC.1396.545) and written informed consents were obtained from parents/guardians of all participants. All methods were performed in accordance with the relevant guidelines and regulations. This multi-center cross-sectional included patients referred to the target hospitals in Tehran, Shiraz, Hamedan and Sari provinces, Iran, from November 14, 2016 to November 15, 2017. The inclusion criteria were age of 1 month to 15 years and suspected meningitis. Children were suspected of bacterial meningitis if the following were present:

- Sudden fever onset (anal temperature > 38.5 °C or axillary temperature > 38 °C); and
- Bulging fontanels, Kerning or Burdzinski signs, neck stiffness, or clinical diagnosis of meningitis.

The exclusion criteria were increased intracranial pressure, localized infection, or bleeding at the site of lumbar puncture. CSF samples were collected through lumbar from all patients. Also, random venous samples were taken from all participants.

### DNA extraction, bacterial identification and typing using multiplex PCR

A volume of 100 μL from each CSF or blood sample were subjected to DNA extraction using QIAamp DNA mini kit (Germany) according to the manufacturer’s instructions [17]. DNA samples were kept in the laboratory of Pediatric Infectious Research Center (PIRC) at a temperature of − 80 °C until multiplex real-time PCR for detection of *S. pneumoniae* and *H. influenzae* were performed. A 20 μL volume-based multiplex real-time PCR using 10 mL master mix (Ampliqon, Denmark), 0.6 pmol/mL of each primer (previously published *ply* for *S. pneumoniae* and *bexA* for *H. influenzae* [18]) and 100 ng of the DNA sample were used. The bacteria were identified in a 95 °C cycle for 5 min, 35 cycles of 95 °C for 25 s, and 69 °C for 60 s. *N. meningitidis* ATCC 13090, *H. influenzae* ATCC 49766 and *S. pneumoniae* ATCC 49619 were considered positive controls.

After confirmation of the pneumococci presence in the samples, 33 serotypes were examined using the multiplex PCR method with previously described specific primer pairs and condition for serotyping of 1, 3, 4, 5, 6A/B, 6C, 7F/A, 7C, 8, 9N/L, 9V, 10A, 11A, 12, 14, 15A, 15 B/C, 16F, 17F, Sg18, 19A, 19F, 20, 22F, 23A, 23B, 23F, 31, 33F, 34, 35B, 35F, 38 [19–23].

### Statistical analysis

The results were reported as positive/negative and the percentages of each serotype were reported in the studied population.

## Results

**Fig. 1:**
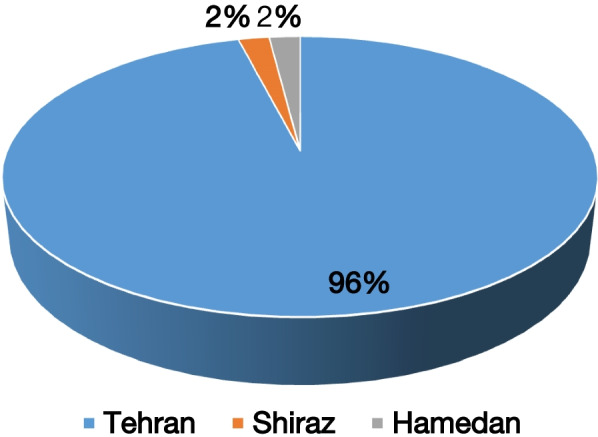
The distribution of CSF samples in different provinces of Iran

Overall, 106 samples were from the target hospital in Tehran (Mofid Hospital), 48 from Hamedan (Besat Hospital), 150 from Shiraz (Namazi Hospital), and 106 from Sari (Booali Sina Hospital). Out of 284 patients suspected of meningitis referred to the target hospitals, all had CSF specimens while only 128 (45%) had blood samples. Of the blood samples, none were positive for *S. pneumoniae* or *H. influenzae*. However, out of 284 CSF samples, 22 (7.7%) were positive for *ply S. pneumoniae*. Out of the 22 samples positive for *ply* gene, two did not meet the Quality Control (QC) standards for DNA extraction. Of the 20 acceptable DNA samples positive for the *ply* gene, 7 were positive for serotype 3, 6 for 11A, 2 for 6A, 2 for 7C, 2 for 14, 1 for 23F, 1 for 23B, 1 for 19A, 1 for 19F and 1 for 5 (Table 1). Nine samples were positive for more than one serotype, with 3 and 11A as the most common combination. In addition, a new co-infection of serotypes 7C and 14 were observed in one sample. Four samples were not typable. The distribution of samples from different provinces are shown in Fig. 1.

**Table 1:** Serotype and sequence types of *ply* positive samples

Number	Sample code	Serotype	Province	Age
1	P40	3, 11A	Tehran	5 years
2	P41	3, 11A	Tehran	4 years
3	P42	3, 11A	Tehran	2 years
4	P46	3, 11A	Tehran	5.5 years
5	P48	3, 11A	Tehran	9 months
6	P49	11A	Tehran	2 years
7	Diana	3, 5A	Tehran	12 months
8	2 S CSF	NT	Tehran	3 years
9	30 H CSF	NT	Hamedan	10 years
10	2M CSF	7C, 14	Tehran	5 years
11	Be	6A, 7C	Tehran	2 years
12	29S	19F, 23B	Tehran	2.5 years
13	16S	NT	Tehran	1.5 years
14	BC	3	Tehran	3 years
15	BCC	6A	Tehran	6 years
16	15S	19A	Shiraz	6 months
17	6S	14	Tehran	5 years
18	10S	23F	Tehran	4 years
19	7S	NT	Tehran	2 years
20	P43	–	Tehran	3 years

Furthermore, we compared patients with and without co-infection regarding age and found that the mean age of patients with co-infection was 3.08 ± 1.82 years, while that of patients without co-infection was 3.64 ± 2.63 years (P = 0.600).

## Discussion

In this study, 412 blood or CSF samples of children with meningitis were examined for *S. pneumoniae* and *H. influenzae* using multiplex PCR. None of the blood samples were positive for the aforementioned organisms and all of the positive samples in this study were CSF specimens. This indicates of the preference of CSF to blood samples for nucleic acid amplification techniques, potentially due to the higher bacterial DNA content of CSF in meningitis. In the current study, 22 CSF samples were positive for *ply S. pneumoniae*. In a study conducted by Amin et al*.* on 196 CSF samples from children (mean age 23 ± 0.56 months), 3 samples were positive for *H. influenzae*, 5 for *S. pneumoniae* and only 1 was positive for *S. agalactiae*, while none of the samples were positive for *N. meningitides* [24]. *H. influenzae* was only identified in 6 samples in the present study.

The main cause of bacterial meningitis in Iran is *S. pneumoniae* with at least 98 known serotypes representing different potentials to cause Invasive Pneumococcal Diseases (IPDs) [25, 26]. Identification of serotypes in patients and finding their patterns of distribution in populations is necessary to determine the required vaccines and the therapeutic protocols [27], while attempts to find an appropriate pattern for *S. pneumoniae* infection still persists. Previous studies reported diverse results for serotypes in IPD patients and also for carriers, which differ from the results of this study. In a previous study, Attarpour et al*.* showed that 18C (44%), 14 (17%), 19A (13%) and 6A (9%) serotypes were the most common serotypes of *S. pneumoniae* causing bacterial meningitis [28]. In another study conducted by Houri et al*.* on children (less than 5 years of age) suspected of IPDs in Tehran, Iran, 23F (24.5%), 19F (18.9%), 19A (7.5%), and 9V (7.5%) serotypes were reported as the most prevalent, respectively [29]. On the other hand, results of studies on nasopharyngeal carriers of *S. pneumoniae* were more similar. In this regard, Ghazikalayeh et al*.*’s results from nasopharyngeal swabs obtained from healthy school students showed the most common serotypes as 19F (30%), 6A/B (18.9%), 15A (16.5%), 11 (11.3%), 23F (8.2%), 1 (6.2%), 19A (3.4%), and 35B (2.4%), respectively [30]. Results of the current study indicated that 3 (35%), 11A (30%) and 6A (10%) serotypes were the most common among the studied patients. Similar results have been presented in a study by Mousavi et al*.* in 2013, in which the results of nasopharyngeal swabs and clinical specimens of children indicated a high distribution of 19A, 6, 3 and 23F serotypes [31]. Differences in demographic features of the study population, variability of the obtained samples, as well as different geographical areas and selected methods for serotyping can be the main reasons for the differences among studies.

Interestingly, this is among the pioneer studies in Iran to report co-infection with dual *S. pneumoniae* serotypes. Sporadically, carriers may have more than one serotype; however, IPD is rarely caused by more than one serotype simultaneously [28]. To the best of our knowledge, only one study has described IPD co-infection with different serotypes of *S. pneumoniae* [28]. In this study, IPD co-infection was associated with age < 5 years and underlying illnesses other than human immunodeficiency virus. Despite extensive knowledge of the pathogenesis microorganisms causing bacterial meningitis, the availability of antibiotics, and the capacity to immunize against these bacteria, bacterial meningitis continues to be a major global source of morbidity and mortality [32]. Infants and young children are particularly at risk for developing bacterial meningitis with under-fives accounting for roughly half of all recorded cases [33]. The results of the current study are consistent with these reports as the mean age of the patients with and without co-infection was under five. However, there was no significant difference between groups.

Ndlangisa et al*.* showed that in 0.1% of samples, one or both isolates were a pneumococcal conjugate vaccine (PCV13) serotype [34]. In our study, 5% of samples showed co-infection with two different serotypes of *S. pneumoniae*. In Ndlangisa et al*.*’s study [28], IPD co-infection with more than one serotype was identified in 35 (0.1%) patients with available viable isolates. In two cases co-infection of serotype 14 with other serotypes (18C and 19A) was observed, but in our study the co-infection was with serotype 14 and 7C. Noteworthy, serotype 14 is included in the PCV13 [28]. Different study populations, and various serotypes in different geographical areas can be responsible for the different co-infection rates and serotypes in their study and ours. The identification of co-infections is of great significance because it can impact the treatment strategy and the infected individual might not respond to routine treatments. This cannot be achieved unless serotyping is performed on the specimens. Furthermore, PCV vaccination has been implemented in Iran since 2010. However, response to vaccines can vary in different individuals, which might be another reason for co-infection.

The major limitation of the current study was that *ply* gene is not specific to *S. pneumoniae*. We could not run PCR for *piaB* and *lytA* genes, which are more specific due to limited resources [35]. Also, the *bexA* gene used for *H. influenzae* in this study encodes the capsulation-associated BexA protein, which is present in all capsulated strains (36); therefore, types e and f could have been missed. Moreover, we could not determine the *H. influenzae* serotypes. Another limitation was that the immune status of the individuals was not available. The sample with co-infection could have been from an immunocompromised patient.

## Conclusion

Co-infection with *S. pneumoniae* serotypes can occur in bacterial meningitis and it might be missed if all serotypes are not evaluated in CSF specimens. We were able to confirm co-infection because all possible sets of multiplex PCRs were carried out for all strains and their serotypes. Therefore, checking for all *S. pneumoniae* serotypes is recommended in CSF specimens from bacterial meningitis.

## Data Availability

The datasets used and/or analyzed during the current study are available from the corresponding author on reasonable request.
